# Expression of sulfotransferase SULT1A1 in cancer cells predicts susceptibility to the novel anticancer agent NSC-743380

**DOI:** 10.18632/oncotarget.2814

**Published:** 2014-11-16

**Authors:** Xiao Huang, Mengru Cao, Li Wang, Shuhong Wu, Xiaoying Liu, Hongyu Li, Hui Zhang, Rui-Yu Wang, Xiaoping Sun, Caimiao Wei, Keith A. Baggerly, Jack A. Roth, Michael Wang, Stephen G. Swisher, Bingliang Fang

**Affiliations:** ^1^ Department of Thoracic and Cardiovascular Surgery, The University of Texas MD Anderson Cancer Center, Houston, Texas, USA; ^2^ Department of Leukemia, The University of Texas MD Anderson Cancer Center, Houston, Texas, USA; ^3^ Department of Laboratory Medicine, The University of Texas MD Anderson Cancer Center, Houston, Texas, USA; ^4^ Department of Biostatistics, The University of Texas MD Anderson Cancer Center, Houston, Texas, USA; ^5^ Department of Bioinformatics and Computation Biology, The University of Texas MD Anderson Cancer Center, Houston, Texas, USA; ^6^ Department of Lymphoma, The University of Texas MD Anderson Cancer Center, Houston, Texas, USA; ^7^ The Fourth Department of Medicine Oncology, Harbin Medical University Cancer Hospital, Harbin, China

**Keywords:** Cancer, drug development, biomarker, sulfotransferase, SULT1A1, personalized therapy

## Abstract

The small molecule anticancer agent NSC-743380 modulates functions of multiple cancer-related pathways and is highly active in a subset of cancer cell lines in the NCI-60 cell line panel. It also has promising *in vivo* anticancer activity. However, the mechanisms underlying NSC-743380's selective anticancer activity remain uncharacterized. To determine biomarkers that may be used to identify responders to this novel anticancer agent, we performed correlation analysis on NSC-743380's anticancer activity and the gene expression levels in NCI-60 cell lines and characterized the functions of the top associated genes in NSC-743380–mediated anticancer activity. We found sulfotransferase SULT1A1 is causally associated with NSC-743380's anticancer activity. SULT1A1 was expressed in NSC-743380–sensitive cell lines but was undetectable in resistant cancer cells. Ectopic expression of SULT1A1 in NSC743380 resistant cancer cells dramatically sensitized the resistant cells to NSC-743380. Knockdown of the SULT1A1 in the NSC-743380 sensitive cancer cell line rendered it resistance to NSC-743380. The SULT1A1 protein levels in cell lysates from 18 leukemia cell lines reliably predicted the susceptibility of the cell lines to NSC-743380. Thus, expression of SULT1A1 in cancer cells is required for NSC-743380's anticancer activity and can be used as a biomarker for identification of NSC-743380 responders.

## INTRODUCTION

Recent advances in the molecular characterization of various cancers have shown that cancers derived from the same origins and with the same histopathological diagnoses and clinical stages can be subgrouped based on their genetic and epigenetic alterations [1-3]. Evidence has shown that cancer is caused by aberrations in the signaling pathways that govern cell proliferation and differentiation, cell survival, and genome stability because of genetic and epigenetic alterations of “cancer driver” genes [4]. These molecular insights into carcinogenesis have led to the successful development of pathway-targeted anticancer therapies, resulting in substantial improvement in clinical outcomes in a subset of cancer patients [5, 6]. However, most such pathway-targeted therapies benefit only a limited number of patients because of the low occurrence frequencies of genetic alterations in the therapeutic targets [7-9]. Consequently, the success of targeted anticancer therapy depends in large measure on biomarkers that can identify the patient subgroups who may respond to the therapeutic agent. Indeed, the inability to identify patient responders is one of major challenges in anticancer drug development, not only causing a failure to demonstrate the potential benefit of a promising anticancer agent [10, 11] but also exposing patients to the risks of ineffective treatment. This was exemplified by the discovery that overexpression or mutation in epidermal growth factor receptors is associated with response to trastuzumab [12, 13], gefitinib [14-16], or erlotinib [14] and can be used for patient selection in the treatment of breast or lung cancers. It is noteworthy that both gefitinib [10] and erlotinib [17, 18] failed to show a benefit in randomized phase III trials with unselected patient populations. Thus, a reliable predictive biomarker is essential to the success of anticancer drug development.

We have recently developed a novel anticancer agent, designated NSC-743380, through chemical library screening of isogenic cells with or without a mutant *KRAS* gene [19] and through lead compound optimization [20-22]. Mechanistic characterization revealed that NSC-743380 and its analogues induced apoptosis in sensitive cancer cells [19-21], inhibited phosphorylation of RNA polymerase II [22, 23], induced sustained JNK activation by inhibiting its dephosphorylation [21], induced reactive oxygen species (ROS) accumulation [24], inhibited STAT3 phosphorylation, and suppressed cyclin D1 expression [20], suggesting that these compounds modulate multiple cancer-related targets. NSC-743380 is highly active (median growth inhibitory concentration [IC_50_] between 10 nM and 1 M) *in vitro* in 30 of 102 cancer cell lines tested [20, 25], including many *KRAS* mutant cancer cells [19, 21, 25]. *In vivo* studies showed that NSC-743380 can induce complete tumor regression or significant growth suppression in several xenograft tumor models at doses that did not cause noticeable adverse effects, demonstrating a wide safety margin and the strong possibility of advancing this agent to clinical trials [20, 25].

Nevertheless, although the lead compound was identified through synthetic lethality screening using *KRAS* mutant cells [19], the anticancer activity of NSC-743380 in the NCI-60 cell panel and in 50 human non–small cell lung carcinoma cell lines did not show a significant correlation with *KRAS* mutations, because a substantial number of *KRAS* wild-type cancer cells were also highly susceptible to NSC-743380 [20, 25]. Therefore, identifying a biomarker that can predict treatment response to NSC-743380 will be critical for future translation into clinical application. To this end, we performed correlation analysis on the IC_50_ values of NSC-743380 in NCI-60 cancer cell lines and levels of mRNA in those cell lines and determined the causal relationship of the candidate genes in NSC-743380–induced anticancer activity. Our results demonstrated that NSC-743380's antitumor activity is dependent on the expression of a sulfotransferase (SULT), SULT1A1, a biotransformation enzyme that bioactivates a number of procarcinogens [26-31].

## RESULTS

### Association of NSC-743380 anticancer activity and gene expression levels in NCI-60 cell lines

**Figure 1:**
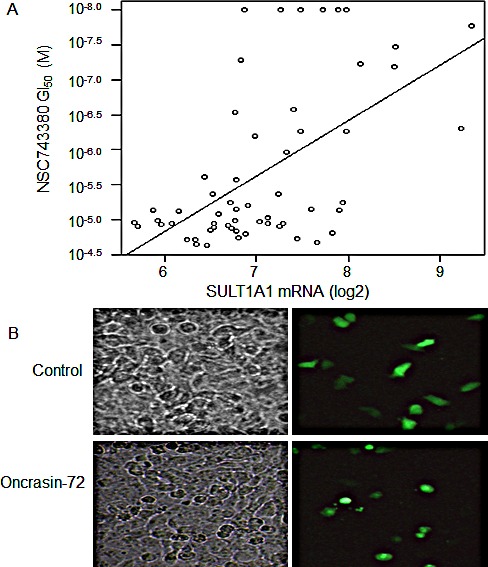
Correlations between SULT1A1 expression and NSC-743380's anticancer activity A) Scattered plot for correlations of NSC-743380' 50% growth inhibition concentrations (GI_50_) and SULT1A1 mRNA levels in NCI-60 cell lines (*r* = 0.56, *p* = 4.13 × 10^−6^). B) Sensitization of H1299 to NSC-743380 by transient transfection with a SULT1A1 expressing plasmid. H1299 lung cancer cells were transfected with plasmids expressing GFP and SULT1A1 for 24 hours and then treated with 1 μM NSC-743380 or dimethyl sulfoxide (DMSO) for 12 hours. The panel shows cell morphology under regular and fluorescent microscopes. Transfection with SULT1A1 alone did not induce cell death (see DMSO group), whereas NSC-743380 induced cell death in transfected cells only (green cells in the treated group).

We previously reported the anticancer activity of NSC-743380 in NCI-60 cancer cell lines and showed that NSC-743380 is highly active in a subset of these lines [20]. To identify biomarkers that can be used to predict response to NSC-743380–induced anticancer activity, we performed Spearman rank tests and Pearson correlation tests to assess whether there were correlations between anticancer activity (−log10 GI_50_) and mRNA levels based on Affymetrix U133A chips (downloaded from the NCI Molecular Target Database, http://discover.nci.nih.gov/cellminer/loadDownload.do). A false discovery rate (FDR) of 5% was used to select genes whose mRNA levels were significantly correlated with NSC-743380's antitumor activity. At FDR of 5%, only SULT1A1 was selected to correlate with NSC-743380's anticancer activity (*r* = 0.56, *p*=4.13 ×10^−6^) (Fig. 1A).

### SULT1A1 is causally associated with NSC-743380–induced antitumor activity

To determine whether SULT1A1 expression has a causal relationship with NSC-743380 susceptibility, we transfected NSC-743380 resistant H1299 lung cancer cells with a plasmid expressing SULT1A1 together with a plasmid expressing GFP. Cells were then treated with 1 μM NSC-743380 to test whether GFP-expressing sensitive cells became resistant or resistant cells became sensitive. Although transfecting the H1299 cells with the SULT1A1-expressing plasmid or treating the cells with NSC-743380 alone did not change the cell morphology, treatment of SULT1A1-transfected H1299 cells induced cell killing of the transfected cells only and not non-transfected cells (Fig. 1B). With the same approach, we tested 10 additional genes on the top list of correlations based on Pearson correlation analysis but was not selected based on FDR of 5%, including BAIAP2, EBI3, REST, CLSTN2, EIF4G3, and BMI1. Constitutively active STAT3 and AKT1 were also included in the study because our previously studies implicated that STAT3 partially contributes to NSC-743380 induced anticancer activity [20]. However, the results showed that none of those genes test could sensitize the H1299 cells to NSC-743380, or cause resistance in NSC-743380 sensitive kidney cell line A498 (data not shown).

**Figure 2:**
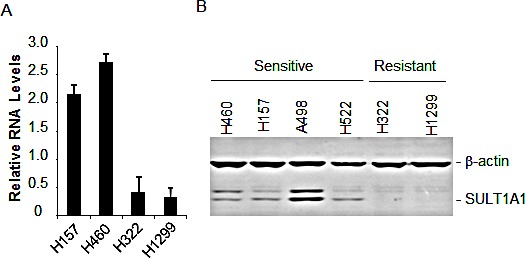
SULT1A1 expression and NSC743380-induced antitumor activity A) mRNA levels in the NSC-743380 sensitive H157 and H460 and NSC-743380 resistant H322 and H1299 cell lines determined by qPCR and normalized with GAPDH as the internal control. The values represent mean +SD of two duplicated assays. B) Western blot analysis of SULT1A1 in NSC743380-sensitive and -resistant cell lines as indicated. β-Actin was used as loading control.

We then performed quantitative PCR analysis on SULT1A1 mRNA in two sensitive (H460 and H157) and two resistant (H322 and H1299) lung cancer cell lines. The results showed that SULT1A1 is highly expressed in the sensitive cells but barely detectable in the resistant cells (Fig. 2A). Western blot analysis also showed that all four sensitive cell lines (H460, H157, A498, and H522) had a clear band of SULT1A1 that was not detectable in the resistant cells (Fig. 2B), consistent with the association between NSC-743380's activity and SULT1A1 mRNA levels observed in the NCI-60 cell lines.

### SULT1A1 is required for NSC-743380–induced antitumor activity

**Figure 3:**
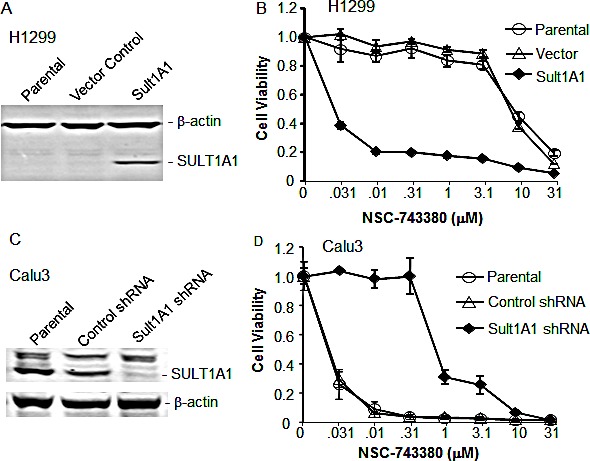
Effect of SULT1A1 overexpression/knockdown on NSC-743380–induced antitumor activity A) Western blot analysis of SULT1A1 in H1299 cells stably infected with retrovirus expressing SULT1A1. B) NSC743380 dose response in parental, vector-transfected, and *SULT1A1*-transfected H1299 lung cancer cells as shown in A. *SULT1A1*-transfected H1299 cells were 1000-fold more sensitive than parental or vector-transfected cells. C) Western blot analysis of SULT1A1 in NSC743380-sensitive Calu3 cells infected with lentivirus expressing scramble or SULT1A1 shRNA. β-Actin was used as loading control. D) NSC743380 dose response of parental, scramble shRNA-transfected, and SULT1A1 shRNA-transfected Calu3 cells as shown in C. SULT1A1 shRNA resulted in resistance to NSC743380 in the Calu3 cells. The values in B and D represent mean ± SD of a quadruplet assay. The assay repeated at least twice with similar results.

To further validate the effect of SULT1A1 in NSC-743380-induced antitumor activity, we performed retrovirus-mediated gene stable overexpression or knockdown analysis. Retroviral vector–mediated stable transfection of *SULT1A1* into H1299 cells rendered the cells highly susceptible to NSC-743380. The IC_50_ values for parental or vector-transfected H1299 cells were >10 μM, whereas in *SULT1A1*-transfected H1299 cells it was about 0.01 μM, a 1000-fold difference (Fig. 3A, B). Interestingly, we found that other major SULT isoforms tested, including SULT1A3, SULT1A4, SULT2A1, and SULT4A1, could not sensitize H1299 cells to NSC-743380 (data not shown), suggesting that SULT1A1 is relatively specific for NSC-743380's anticancer activity. Moreover, knockdown of SULT1A1 in Calu3 cells by retrovirus-mediated shRNA expression diminished the sensitivity of those cells to NSC-743380 (more than 100-fold increase in IC_50_) (Fig. 3C, D). These results further demonstrated that SULT1A1 expression is causally associated with NSC-743380–induced antitumor activity.

### SULT1A1 as a predicting biomarker for response to NSC-743380 in leukemia cell lines

**Figure 4:**
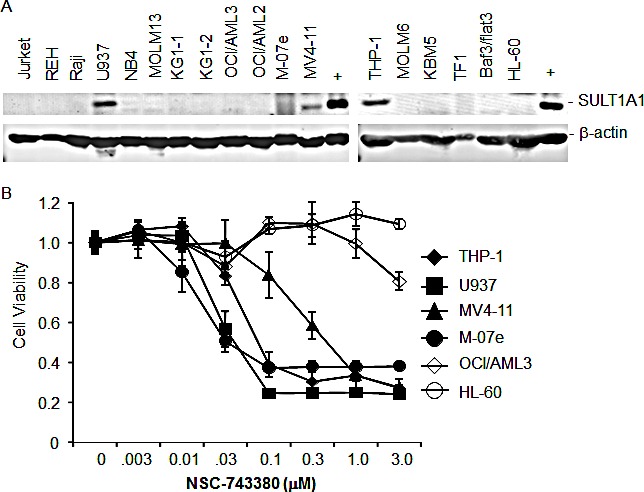
SULT1A1 expression predicts NSC743380-sensitivity in leukemia cell lines A) Expression of SULT1A1 in cell lysates of 18 leukemia cell lines. The cell lysates were harvested several years ago and stored them in a –80°C freezer. Cell lysates from the kidney cell line A498 were used as positive control. The results showed that U937, M07e, MV4-11, and THP-1 were positive for SULT1A1 proteins. β-Actin was used as loading control. B) Cell viability assay for six leukemia cell lines. The cells were treated with various concentrations of NSC-743380. Cell viability was determined 3 days after treatment using the MTS assay. Control cells (indicated by the 0 in the concentration) were treated with solvent (dimethyl sulfoxide), and their value was set as 1. The values represent mean ± SD of a quadruplet assay. The assay repeated at least twice with similar results.

To test whether expression of SULT1A1 in other cancer cells can be used to predict responses to NSC-743380, we used Western blot analysis to determine SULT1A1 expression in cell lysates from 18 leukemia cell lines. Lysates from the A498 kidney cell line, which is highly sensitive to NSC-743380 both *in vitro* and *in vivo* [20], were used as positive control. The Western blot analysis showed that SULT1A1 was expressed in four of the leukemia lines: U937, M-07e, MV4-11, and THP-1 (Fig. 4A). We then performed the cell viability assay on six leukemia cell lines, including the four lines that expressed SULT1A1 and two cell lines (HL-60 and OCI/AML3) that did not. Cells were treated with NSC-743380 at doses ranging from 0.003 to 3 μM for 72 hours, and cell viability was determined by using MTS assay as described previously [32]. The results showed that all four cell lines expressing SULT1A1 were sensitive to NSC-743380, with IC_50_ values between 0.03 and 0.3 μM, whereas the two SULT1A1-negative cell lines were resistant, with IC_50_ >3 μM (Fig. 4B), demonstrating that SULT1A1 expression can be used to identify sensitive cancer cells *in vitro*.

## DISCUSSION

Our study demonstrated that NSC-743380's anticancer activity is causally associated with SULT1A1 expression in cancer cells and that SULT1A1 expression can be used as a biomarker to predict response or identify responders to NSC-743380.

Sulfotransferases are a family of biotransformative enzymes that catalyze the sulfation of numerous xenobiotics, drugs, and endogenous compounds, leading to an increase in the compound's solubility and often a decrease in its biological activity [33]. At least 11 distinct cytosolic SULTs have been identified in humans [33], and some of these enzymes, including SULT1A1, are known to bioactivate compounds such as procarcinogens [26-31]. Because of the role of SULTs in carcinogen-mediated malignant transformation, their overexpression is expected to render a cell more susceptible to malignant transformation by SULT-activated carcinogens and could be a marker for a subtype of carcinogen-induced cancers. Indeed, members of the SULT1A subfamily have been found to be highly expressed in breast cancer cell lines but were not detected in normal human mammary epithelial cells [34]. The expression of SULT1A1 was readily detectable in primary breast cancer tissues but not in neighboring normal tissues [35], and SULT1A1 activity was drastically higher in hepatocellular carcinoma patients [36]. Patients with liver cirrhosis who had higher SULT1A1 activity had a higher risk of developing hepatocellular carcinoma than did such patients with normal SULT1A1 activity. However, there is a huge gap in our knowledge concerning the expression status of SULTs in tumor tissues versus normal tissues, although expression of some SULT isoforms in certain normal human tissues has been reported [37-39]. Several studies have been performed to determine the association between *SULT1A1* polymorphic alleles in blood cells and the risk of breast cancer [40-42], lung cancer [43, 44], colorectal cancer [45, 46], bladder cancer [47], and brain tumors [48], but the results have been inconsistent. Nevertheless, the more active allele of SULT1A1 has been associated with better survival outcome in breast cancer patients treated with tamoxifen [49], possibly because the SULT1A1-mediated biotransformation of 4-hydroxytamoxifen potentiates the efficacy of tamoxifen therapy [35]. SULT1A1 has also been reported to be required for the anticancer activity of aminoflavone, an aryl-hydrocarbon receptor ligand [50], suggesting that SULT1A1 could play an important role in anticancer therapy. Together, the results from this study and the studies on tamoxifen [49] and aminoflavone [50] suggest that intratumoral expression of SULT1A1 may serve as a bioactivator for some anticancer agents and as a biomarker to identify responders to those therapeutics in a subgroup of cancer patients.

NSC743380 is derived from the lead compound oncrasin-1, which was identified through synthetic lethality screening on isogenic cells with or without a mutant *KRAS* gene [19]. Activating mutations in oncogenic *RAS* genes are among the first and the most common genetic alterations identified in human cancers [51, 52]. Extensive efforts have been made to develop therapeutics targeting to RAS signaling pathways [6, 52], however, effective anti-RAS therapeutics is not yet clinically available. Our previous studies revealed that NSC743380 is highly active in a number of *KRAS* mutant cancer cell lines [20, 25]. Nevertheless, the correlations between NSC-743380's anticancer activity and *KRAS* mutations in the NCI-60 cell lines and in the 50 tested lung cancer cell lines were not significant [20, 25]. A possible explanation is that *KRAS* mutant cancer cells can be categorized as *KRAS*-dependent and *KRAS*-independent [53], and that RAS activation signatures are observed in substantial numbers of RAS wilt-type tumors [54]. Moreover, cancer cells with different *KRAS* mutations may have different metabolic profiles [55]. Interestingly, *KRAS* mutations are more common in smoking-associated cancers [56-58]. Intriguingly, SULT1A1 is capable of bioactivating procarcinogens [26-31] and likely plays a role in tobacco-induced carcinogenesis. Whether oncogenic *KRAS* is involved in regulating SULT1A1 expression, or whether SULT1A1 overexpression promotes smoking-induced *KRAS* mutations, remains to be determined. Nevertheless, the fact that KRAS transfected cells, but not the parental cells, were highly susceptible to oncrasin-1 and NSC-743380 [19, 22] indicates that KRAS may upregulate SULT1A1 in some cancer cells, and that SULT1A1 overexpression may occur in a subset of cancers with activation of RAS signaling pathways. The differential expression of SULT1A1 in tumor tissues versus neighboring normal tissues [35] suggests that expression of SULT1A1 in tumors, but not the germ line SULT1A1 haplotypes, is a more appropriate biomarker to identify responders for therapeutics activated by SULT1A1. Our results strongly suggest that expression levels of some procarcinogen-activating enzymes in cancers can be exploited as a biomarker for identifying responders of some anticancer agents.

## Methods

### Cell lines and cell culture

The human non–small cell lung carcinoma cell lines were routinely grown in Dulbecco's modified Eagle's medium supplemented with 10% fetal bovine serum and 100 μg/mL penicillin-streptomycin (all from Life Technologies), as previously described [25]. Cells were cultured at 37°C in a humidified incubator containing 5% CO_2_. Leukemia cell lines were obtained from the American Type Culture Collection. The culture conditions were the same as above. The cell lines were regularly authenticated with short tandem repeat fingerprint method.

### Chemicals and reagents

NSC-743380 was synthesized as previously described [22]. Antibody against SULT1A1 was obtained from R&D Systems and antibody for β-actin from Sigma. The pEGFP-N1 plasmid for expressing green fluorescent protein (GFP) was obtained from Clontech. The plasmids expressing constitutively active STAT3 (STAT3CA) and AKT1 (AKT1CA) were described previously [20, 59]. All other plasmids expressing cDNAs or short hairpin RNA (shRNA) were obtained from either Origene Technologies or Open Biosystems. All plasmids expressing cDNAs were verified by DNA sequencing performed at the Sanger DNA Sequencing Core Facility at our institution.

### Plasmid transfection and retrovirus infection

Plasmid transfection was performed using the FuGENE6 reagent (Promega). Cells were transfected with a plasmid encoding a gene to be tested and pEGFP-N1 at a ratio of 1:1 for 24 hours and then treated with 1 μM NSC-743380 overnight. The morphology of transfected cells was observed under the fluorescent microscope. For establishing stable gene expression or knockdown, retrovirus or lentivirus was produced in 293/Phoenix cells and used for infecting cells as described previously [21]. Stable transfectants were selected for growth in the presence of 500–800 μg/ml G418 (Geneticin) or 1–5 μg/ml puromycin, based on the selection marker in the vector backbones. The selected cells were pooled together for the studies.

### Cell viability assay

Cell viability for monolayer cells was determined by using the sulforhodamine B (SRB) assay as we described previously [21]. Cell viability for suspension cell cultures was determined by using [3-(4,5 dimethylthiazol-2-yl)-5-(3-carboxymethoxyphenyl)-2-(4-sulfophenyl)-2H-tetrazolium (MTS) as described previously [32]. Each experiment was performed in quadruplicate and repeated at least three times. The IC_50_ valuewas determined by using the CurveExpert Version 1.3 program.

### Real-time PCR assay

Total RNA was extracted from cells using the Trizol reagent (Invitrogen). Reverse transcription and real-time PCR were performed as we previously described [21]. The following primers were used for real-time PCR: SULT1A1, sense 5′-ACTGGAAGACCACCTTCACC-3′, antisense 5′-GTCAGGTTTGATTCGCACAC-3′; GAPDH, sense 5′-GGCTCTCCAGAACATCATCC-3′, antisense 5′-TAGCCCAGGATGCCCTT-3′. The primers for the target gene *SULT1A1* were confirmed to have amplification efficiency equal to that of the reference gene GAPDH. The relative RNA expression was calculated automatically by the installed software of the instrument with the ΔΔCt method, using GAPDH as a reference gene.

### Western blot

Cells were harvested and subjected to lysis in Laemmli lysis buffer. The protein concentration was determined using the Bradford method. Equal amounts of lysates (40 μg) were separated by 10% sodium dodecyl sulfate–polyacrylamide gel electrophoresis (SDS-PAGE) and then transferred to Hybond-enhanced chemiluminescence membranes (Amersham Corp.). Membranes were then blocked with PBS buffer containing 5% low-fat milk and 0.05% Tween (PBST) for 1 hour and then incubated with primary antibodies overnight at 4°C. After being washed three times with PBST, membranes were incubated with peroxidase-conjugated secondary antibodies for 1 hour at room temperature. The membranes were washed with PBST again and developed with a chemiluminescence detection kit (ECL kit, Amersham Bioscience). β-actin was used as a loading control.

### Statistical analysis

Differences between treatment groups were assessed using the unpaired Student's *t* test at a significance level of *P* < 0.05. For analysis of correlations between gene expression and NSC-743380's activity in the NCI-60 cell lines, the Affymetrix U133A and U133B gene expression microarray data were downloaded from the National Cancer Institute website (http://discover.nci.nih.gov/cellminer/loadDownload.do). The expression levels were quantified using the Robust Multiarray Analysis method. Spearman rank tests (not assuming normality) and Pearson correlation (assuming normality) were used to assess whether there were associations between anticancer activity and gene expression. A beta-uniform mixture (BUM) model [60] was used to estimate the false discovery rate (FDR).
